# Effectiveness of home-based conventional exercise and cryotherapy on daily living activities in patients with knee osteoarthritis: A randomized controlled clinical trial

**DOI:** 10.1097/MD.0000000000033678

**Published:** 2023-05-05

**Authors:** Hawar Abdulrazaq MohammedSadiq, Mohammad Tahir Rasool

**Affiliations:** a Adult and Fundamentals of Nursing Unit, College of Nursing, University of Duhok-Iraqi Kurdistan, Duhok, Iraq; b Department of Medicine, College of Medicine, University of Duhok-Iraqi Kurdistan, Duhok, Iraq.

**Keywords:** joint stiffness, pain, physical function

## Abstract

Knee osteoarthritis (KOA) is a prevalent joint condition associated with aging that causes pain, disability, loss of function, and a decline in quality of life. This study aimed to evaluate the effectiveness of home-based conventional exercise and cryotherapy on daily living activities in patients with KOA.

**Methods:**

In this randomized controlled clinical trial, the patients who were diagnosed with KOA were assigned to 3 groups: an experimental group (n = 18), the control group 1 (n = 16), and the control group 2 (n = 15). Control and experimental groups engaged in a 2-month home-based exercise (HBE) program. The experimental group received cryotherapy along with HBE. In contrast, the patients in the second control group received regular therapeutic and physiotherapeutic services at the center. The patients were recruited from the Specialized Center for Rheumatic and Medical Rehabilitation in Duhok, Iraq.

**Results:**

The patients in the experimental group had statistically significant better daily activity functions compared to the first and second control groups in pain (2.22 vs 4.81 and 12.7; *P* < .0001), stiffness (0.39 vs 1.56 and 4.33; *P* < .0001), physical function (5.72 vs 13.31 and 38.13; *P* < .0001), and the total score (8.33 vs 19.69 and 55.33; *P* < .0001) at 2 months. The patients in the experimental and the first control groups had statistically significantly lower balance scores compared to the second control group at 2 months, 8.56 versus 9.30. At 3 months, similar patterns were observed for the daily activity function and balance.

**Conclusions:**

This study showed that combining HBE and cryotherapy may be an effective technique to improve function among patients with KOA. Cryotherapy could be suggested as a complementary therapy for KOA patients.

## 1. Introduction

Knee osteoarthritis (KOA) is a prevalent joint condition associated with aging that causes pain, loss of function, and disability, as well as a decline in quality of life, with a significant societal and economic cost.^[1]^ It is considered to be the main cause of disability in the elderly. The systematic reviews have reported that the pooled global prevalence of KOA is 22.9% (95% CI: 19.8%–26.1%) among persons aged 40 years and older. It is estimated that close to 654.1 (95% CI: 565.6–745.6) million persons aged 40 years and older have been affected by this disease globally. The rate of this disease is different between countries and increases with age. The reported ratios of prevalence and incidence among female and male populations are 1.69 (95% CI: 1.59–18.0, *P* < .0001) and 13.9 (95% CI, 1.24–1.56: *P* < .0001), respectively.^[2]^

Disease prevalence varies by geographical location. In the Middle East and North Africa, 24.6 million cases (95% UI: 22.0–27.3 million) of osteoarthritis (OA), with an age-standardized point prevalence of 5342.8 per 100,000 (95% UI: 4815.9–5907.8) were recorded, which was 9.3% higher than in 1990 (95% UI: 8.1–10.5%).^[3]^ The literature has shown that women over 50 are more likely than men of the same age to develop OA.^[4]^

The most common type of OA is KOA.^[5]^ Improving the quality of life is key to controlling KOA symptoms. Symptoms can be relieved with pharmacological treatment. However, some of the most recommended medications are poorly tolerated and cause systemic adverse effects with long-term use.^[6,7]^ Therefore, as there is no cure for OA, it is vital to employ non-pharmacological therapy to limit its development, alleviate symptoms, and enhance knee function and quality of life.^[8]^ Current clinical practice guidelines recommend a combination of pharmacological^[9]^ and non-pharmacological treatments.^[10]^ Studies demonstrate that a simple home-based exercise (HBE) program improves KOA patients’ strength and function while reducing pain.^[11]^ However, the effects of HBE in conjunction with cryotherapy have yet to be examined on patients with KOA. Therefore, we need more reports in this regard.

Cryotherapy, a non-pharmaceutical treatment, has been widely used to treat some rheumatic joint conditions.^[12,13]^ It can be used alone or in conjunction with other therapies depending on its impact on pain, swelling, and inflammation.^[13]^ Although some international KOA guidelines recommend cryotherapy as a treatment option,^[14–16]^ others have found insufficient evidence to support it.^[17,18]^ Relevant systematic reviews have also indicated that further data with better methodological rigor is required to evaluate the effects of cryotherapy on pain, function, and quality of life in patients with KOA.^[19,20]^ These studies have also reported significant limitations, including uncovered allocation, a lack of blinding, inadequate baseline group comparability, a lack of confirmation of the participants’ OA grade, and not adhering to the intention-to-treat principle.^[19]^ Therefore, more attempts are required to meet these methodological standards. In this study, we aimed to evaluate the effectiveness of a program of HBE and cryotherapy on the daily living activities of patients with KOA. We hypothesized that a program of HBE and cryotherapy combined would more effectively relieve patients’ symptoms at week 8, including pain and stiffness, and improve daily functional activities and balance, compared to a program of HBE alone.

## 2. Patients and methods

### 2.1. Study design

In this randomized controlled clinical trial, the patients who were diagnosed with KOA were assigned to 3 groups. The patients in the experimental group engaged in a 2-month program of HBE with cryotherapy. The patients in the first control group received only HBE for 2 months, while those in the second control group received regular therapeutic and physiotherapeutic services at the center.

**Figure 1.**
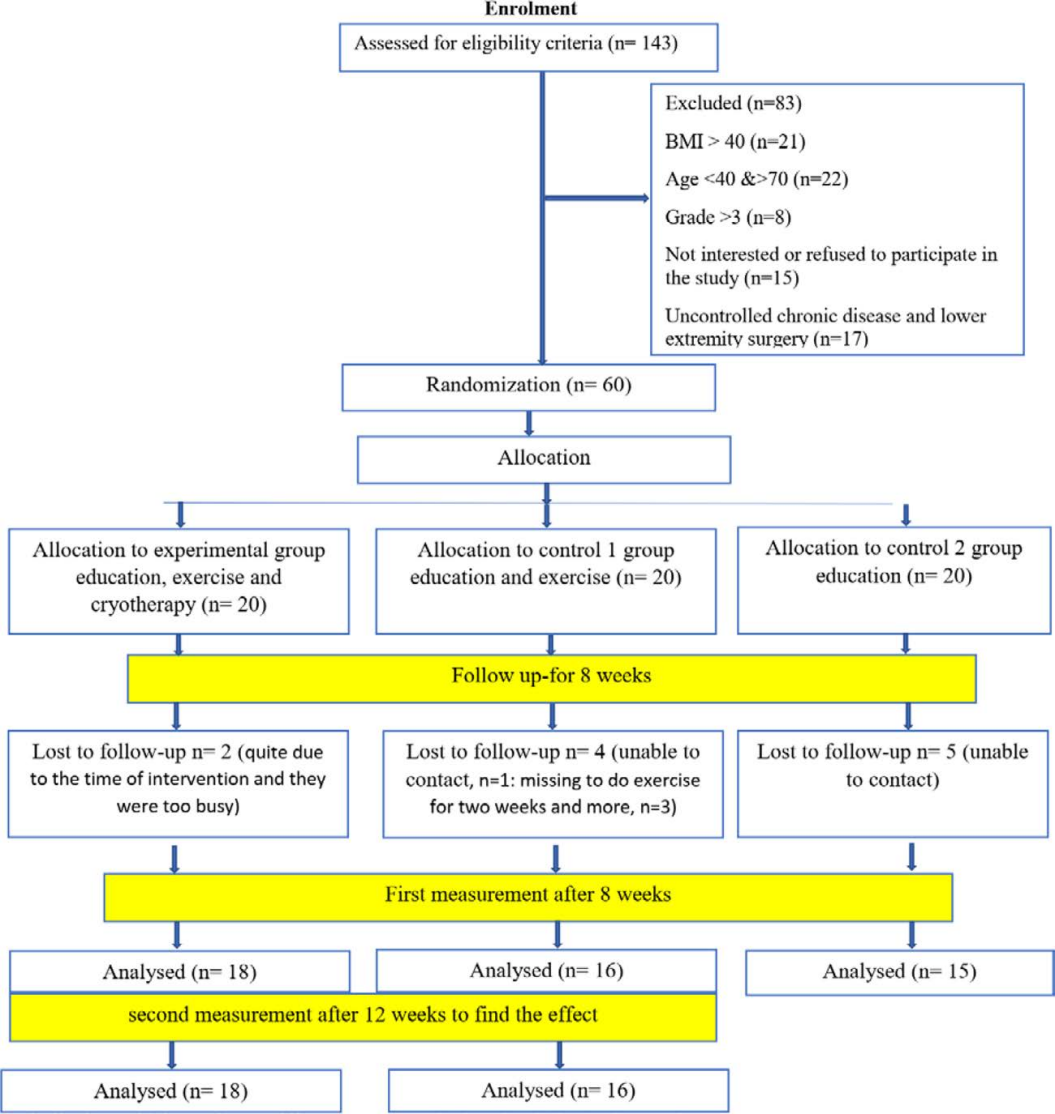
Flowchart of patients’ recruitment.

The patients in all 3 groups received education about the risk factors of KOA based on the center regulations. The patients who attended the center were screened medically and clinically for eligibility. Those diagnosed with KOA by a rheumatologist in the center met the initial eligibility criteria for this study and were referred to the researchers at the center. The researchers measured the patient body mass index (BMI), checked other eligibility criteria, and received written consent from those who met the eligibility criteria for the next step. The patients were then randomly assigned to the experimental, control, or second control groups (see flow chart in Fig. 1).

The patients were recruited from the Specialized Center of Rheumatic Diseases and Medical Rehabilitation in Duhok City in Iraqi Kurdistan between August 1^st^, 2021, and June 1^st^, 2022. The patients received a 2-month intervention and a follow-up 1 month later. The ethical approval of this study was obtained from the local health ethics committee in Duhok City. The ethics committee is a joint health committee between the University of Duhok and the Duhok General Directorate of Health, registered in reference number 13072021-7-27 on July 13, 2021.

### 2.2. Setting, participation, and sampling

The center the patients were recruited from is the only specialized center in our city for diagnostic and therapeutic services for patients with rheumatoid diseases and rehabilitation services. Before the inclusion of the patients in the study, the researchers obtained general information about the target population, including gender, age, BMI, disease grade, and education of patients, to ensure an appropriate and representative sample of patients in the study. The researchers attended the center twice a week for 6 months to collect data, and the required coordination was made with the clinicians for this study.

The researchers attempted to recruit patients from different clinicians to have a more representative sample of the patients in this region. In addition, the researchers checked the medical and clinical information of the patients to ensure the correct diagnosis. The patients who met the eligibility criteria were assigned to 1 of the 3 groups based on random, pre-generated 3-group numbers. The total number of patients was calculated by sampling and entered into the Statistical Software for Social Sciences 25 (SPSS 25) to obtain a pre-generated 3-group number. The numbers were randomly assigned into 3 groups without giving their names. Then, the groups were randomly allocated to the patients to avoid bias. The center clinicians had no role in the intervention and measurements of outcomes and were blind to the patients’ allocations to the groups in this study.

To prevent allocation bias, the patients were from different geographic areas within the Duhok province. Only 2 patients were from the same family. In this situation, we included the first diagnosed case in the determined group. The second case was given the same intervention but was not included in the study. In addition, to avoid bias, we did not give information about cryotherapy to the control group. We only obtained written consent from the patients for the applied intervention. The researchers followed up with the patients weekly at their houses.

### 2.3. Sample size

The mean values of the patients with KOA were obtained from previous studies. Subsequently, the sample size was obtained according to the expected effect of cryotherapy and HBE on the function of patients with KOA. In this regard, we expected that the mean value of 6.85 (standard deviation [SD]: 1.57) be reached 1.57 (SD: 1.67) considering the large sample size (Cohen d 1.39). The required sample size for each group was 15 patients. The actual power was 0.9562 (two tails, α error prob of 0.05, and allocation ratio N2/N1 of 1). G*Power 3.1.9 was used to calculate the sample size.

### 2.4. Eligibility criteria

The inclusion criteria were: diagnosis of KOA grade ≤3; current joint symptoms; 40 to 70 years of age; experienced knee pain most days of the previous month; knee pain average between 3 and 7 on the visual analog scale for pain in the previous week; and BMI < 40.

Exclusion criteria were: rheumatoid arthritis or other systemic rheumatic diseases; dementia, psychosis, or active substance abuse disorder; acute or chronic disease, injury, deformity, or operation of the lower limb and knee; severe hearing or visual impairment; hospitalization for a cardiovascular condition, cerebral infarction or arrhythmia in the previous 3 months; recent history of 3 or more falls; current participation in another OA intervention study; uncontrolled diabetes mellitus; intra-articular knee injections in the previous 6 months; and contraindication(s) to cryotherapy application (i.e., those that feel a high level of discomfort or pain during the application). The participants signed a written informed consent form, and then the author completed the baseline assessments. The patients who did not meet the eligibility criteria were not given a number to avoid bias.

### 2.5. Outcome measurements

The outcomes of the study were measured after the completion of the follow-up time. The interventions of 3 groups were applied to the patients by the first author (a nurse physiotherapist). The researchers trained a college-graduated nurse to obtain the outcomes’ measurements in all groups to avoid bias. But the trained nurse was masked in allocating the patients into the study groups. The trained nurse performed the pre-and post-intervention measurements.

### 2.6. Pilot study

We performed a pilot study on 5 patients. In this regard, the first 5 cases who were diagnosed with KOA received the interventions as determined in the pre-generated random numbers. The pilot study aimed to check the difficulties and challenges of the study, as the researchers were not sure of the study feasibility. The researchers continued the study when they faced no significant challenges or difficulties. In addition, the reliability of the Western Ontario and McMaster Universities Osteoarthritis Index (WOMAC) scale of the first 5 cases was measured from the pilot study.

### 2.7. Interventions

#### 2.7.1. Experimental and control group 1.

For their first session, the patients in the experimental group received a 30-minute education in their homes to avoid interruptions at the center. Health education covered the concepts of clinical manifestations, risk factors, nursing care for KOA, benefits of HBE, and cryotherapy. The researchers created a video of each exercise and a booklet for the patients (in the Kurdish and Arabic languages), which were given to the patients to ensure they would perform the exercises correctly. The researcher helped the patients to perform the first HBE intervention as outlined in the booklet, and then the remaining exercises were performed by the patients.

The researcher visited the participants’ homes over 8 weeks (weeks 1, 3, 5, and 7) to encourage them to perform the exercises and avoid losing follow-up. Each session included 70 to 75 minutes of exercise and 20 minutes of cryotherapy. Two packs of reusable gel ice packs (measuring 48 × 15 × 0.5 cm) were placed on the knee for 20 minutes, covering the anterior, posterior, medial, and lateral surfaces (See supplementary file, http://links.lww.com/MD/I910 Details of Home-Based Exercises & Cryotherapy for detailed information on the HBE intervention and cryotherapy).

The researcher called participants at weeks 2, 4, 6, and 8 to discuss their progress and to encourage them to continue to adhere to the exercise and cryotherapy program. The study lasted for 8 weeks, with 3 sessions per week occurring on nonconsecutive days, for 24 sessions. It was confirmed that the patients in the experimental group and control group 1 adhered completely to the exercises, as the first author visited their homes or called them continuously to record the adherence. Regarding the second control group, patients received their normal exercises and physiotherapy sessions as usual.

The patients in control group 1 received a similar education and HBE program as the experimental group. However, these patients did not receive cryotherapy. The patients received followed-up attention like those in the experimental group (see supplementary information, http://links.lww.com/MD/I910).

#### 2.7.2. Control group 2.

In the first session, the patients in control group 2 received health education about the risk factors of KOA. The researcher did not visit these patients at home because they received no intervention. However, the researcher called these patients for follow-up purposes and to check their adherence to regular therapy given by clinicians. These patients received routine exercise and education at the center, including hip and knee flexion, short arc quad, straight leg raise, and long arc quad, as described below:

Step 1: Hip and knee flexion:

• Lying straight on your back, bend your knee and hip.
• Your kneecaps must point toward the ceiling.
• Hold your knee in the position for 5 seconds, then return to the start position.

Step 2: Short arc quad

• Place a firm roll or a rolled towel under your knee.
• Straighten the knee to lift your foot off the bed.
• Hold it in this position for 5 seconds. Slowly lower the foot down to the bed.

Step 3: Straight leg raise

• Lie on your back and put 1 leg in a comfortable position.
• Slowly raise the other painful leg, keeping the knee straight.
• Hold the knee in this position for 5 seconds and slowly lower your leg.

Step 4: Long arc quad

• Sitting on a chair with your thigh supported, lift your foot until your knee is completely straight.
• Hold your knee in this position for 5 seconds.
• Slowly return to the starting position and relax.

#### 2.7.3. Assessment of outcomes.

The principal investigator collected the baseline information of the patients and recorded them in a pre-designed questionnaire. This information included age, gender, height, weight, BMI, marital status, educational level, occupation, smoking, disease duration, fall history within the past year, affected knees, grade, analgesics, cartilage drug, and comorbidity. We counted the comorbidities according to the Elixhauser Comorbidity Index. These were congestive heart failure, cardiac arrhythmias, valvular disease, pulmonary circulation disorders, peripheral vascular disorders, hypertension (uncomplicated), hypertension (complicated), paralysis, other neurological disorders, chronic pulmonary disease, diabetes (uncomplicated), diabetes (complicated), hypothyroidism, renal failure, liver disease, peptic ulcer disease (excluding bleeding), AIDS/HIV infection, lymphoma, metastatic cancer, solid tumor without metastasis, rheumatoid arthritis/collagen vascular diseases, coagulopathy, obesity, weight loss, fluid and electrolyte disorders, blood loss anemia, deficiency anemia, alcohol abuse, drug abuse, psychoses, and depression. The complications of both study groups were documented at 2 and 3 months.

#### 2.7.4. Primary outcome measures.

The study primary outcomes were pain intensity, joint stiffness, and physical function, measured by the WOMAC.^[21]^ It includes 5 items on pain, 2 items on joint stiffness, and 17 on physical function, rated on a 0 to 4 Likert scale. Higher scores reflect greater pain, stiffness, and difficulty performing physical functions. The internal reliability of the WOMAC, measured by Cronbach alpha, is 0.67 to 0.82 for its 3 subscales, and its test-retest reliability, based on the interclass correlation coefficient, is 0.82 to 0.88 for its 3 subscales.^[22]^

#### 2.7.5. Secondary outcome measures.

The Timed Up measured the fall risk and progress of balance, sit-to-stand, and walking and Go (TUG) test. The TUG test measures how long participants can rise from a chair of normal height, walk 3 meters, turn, and sit back down.^[23]^

#### 2.7.6. Statistical methods.

The patients’ general information in the 3 groups was presented in mean (SD) or number (%). The comparisons of baseline information of patients with KOA were examined in 1-way ANOVA or Pearson chi-squared tests. Their WOMAC function scores and TUG tests were compared in 1-way ANOVA. The post hoc comparisons were examined in a Tukey test. Comparisons of WOMAC scores of patients with KOA between study steps in the control groups were examined in a Bonferroni test. A significant level of difference was identified in a *P* value < .05. The statistical calculations were performed in JMP Pro 14.3.0.

## 3. Results

### 3.1. Comparisons of baseline information

The participants in the experimental and control groups were similar in baseline information at the intention-to-treat step. The homogeneity of the baseline and medical information was checked between the lost to follow-up patients and study groups to examine the successfulness of the randomization. The patients in the control, experimental, and lost-to-follow-up groups were similar in general and medical information, as shown in Table 1.

**Table 1:** Comparisons of baseline information of patients with knee osteoarthritis among study group (per-protocol approach).

Socio-demographic characteristics			Study groups		*P* value (2-sided)
	Second control (n = 15)	Control (n = 16)		Experimental (n = 18)	
Age	53.8 (8.46)	51.38 (7.72)		51.94 (5.84)	.6331*
Age category					
41–50	7 (46.67)	7 (43.75)		8 (44.44)	.4390†
51–60	4 (26.67)	7 (43.75)		9 (50.00)	
61–70	4 (26.67)	2 (12.50)		1 (5.56)	
Gender					
Female	14 (93.33)	13 (81.25)		13 (72.22)	.2961†
Male	1 (6.67)	3 (18.75)		5 (27.78)	
BMI	34.14 (4.45)	31.61 (4.12)		32.91 (3.95)	.2502*
BMI category					
Normal weight	0 (0.00)	0 (0.00)		1 (5.56)	.6258†
Overweight	4 (26.67)	6 (37.50)		4 (22.22)	
Obese	11 (73.33)	10 (62.50)		13 (72.22)	
Marital status					
Married	12 (80.00)	11 (68.75)		14 (77.78)	.3637†
Single	0 (0.00)	2 (12.50)		0 (0.00)	
Widow	3 (20.00)	3 (18.75)		4 (22.22)	
Education					
Illiterate	10 (66.67)	9 (56.25)		8 (44.44)	.7112†
Institute/College	0 (0.00)	1 (6.25)		0 (0.00)	
Intermediate school	1 (6.67)	2 (12.50)		3 (16.67)	
Primary school	4 (26.67)	4 (25.00)		6 (33.33)	
Secondary school	0 (0.00)	0 (0.00)		1 (5.56)	
Occupation					
Needs mild mobility	2 (13.33)	3 (18.75)		5 (27.78)	.3593†
Needs moderate mobility	13 (86.67)	11 (68.75)		10 (55.56)	
Needs high mobility	0 (0.00)	2 (12.50)		3 (16.67)	
Smoking					
No	14 (93.33)	14 (87.50)		18 (100.00)	.3144†
Yes	1 (6.67)	2 (12.50)		0 (0.00)	
Disease duration	8.67 (4.34)	8.75 (3.57)		11.11 (4.27)	.1499*
Fall history					
No	15 (100.00)	15 (93.75)		18 (100.00)	.3490†
Yes	0 (0.00)	1 (6.25)		0 (0.00)	
Affected knees					
One knee	11 (73.33)	11 (68.75)		15 (83.33)	.5976†
Two knees	4 (26.67)	5 (31.25)		3 (16.67)	
Disease grade					
1	1 (6.67)	0 (0.00)		2 (11.11)	.6570†
2	11 (73.33)	14 (87.50)		14 (77.78)	
3	3 (20.00)	2 (12.50)		2 (11.11)	
Analgesics					
No	1 (6.67)	1 (6.25)		4 (22.22)	.2675†
Yes	14 (93.33)	15 (93.75)		14 (77.78)	
Cartilage drug					
No	9 (60.00)	14 (87.50)		14 (77.78)	.1974†
Yes	6 (40.00)	2 (12.50)		4 (22.22)	
Comorbidity					
No	6 (40.00)	7 (43.75)		11 (61.11)	.4233†
Yes	9 (60.00)	9 (56.25)		7 (38.89)	

BMI = body mass index.

* ANOVA 1-way.

† Pearson chi-squared tests were performed for statistical analyses.

#### 3.1.1. Comparisons of WOMAC function scores at baseline.

The participants in the control and experimental groups were similar at baseline in pain (14.60, 14.13, and 13.17; *P* = .0879), stiffness (4.80, 4.82, and 4.89; *P* = .9620), physical function (41.80, 42.06, and 38.39; *P* = .1218), total function (61.20, 61.00, and 56.44; *P* = .124), and balance (15.50, 15.18, and 14.42; *P* = .5094) (see Table 2).

**Table 2:** Comparisons of WOMAC function scores and TUG test of patients with knee osteoarthritis among study group at baseline.

WOMAC function scores	Study groups			*P* value (2-sided)
	Second control (n = 15)	Control (n = 16)	Experimental (n = 18)	
Pain score	14.60 (1.64)	14.13 (2.09)	13.17 (1.82)	.0879
Stiffness score	4.80 (0.68)	4.82 (1.22)	4.89 (1.02)	.9620
Physical function score	41.80 (5.98)	42.06 (4.68)	38.39 (6.28)	.1218
Total WOMAC score	61.20 (6.58)	61.00 (7.23)	56.44 (8.37)	.1224
TUG	15.50 (2.61)	15.18 (3.62)	14.42 (1.83)	.5094

TUG = timed up and go test, WOMAC = Western Ontario and McMaster Universities Osteoarthritis Index.

ANOVA 1-way was performed for statistical analyses.

#### 3.1.2. Comparisons of WOMAC function scores per-protocol approach.

The study showed that the patients in the experimental group had statistically significantly better daily activity functions compared to the first and second control groups in pain (2.22 vs 4.81 and 12.7; *P* < .0001), stiffness (0.39 vs 1.56 and 4.33; *P* < .0001), physical function (5.72 vs 13.31 and 38.13; *P* < .0001), and the total score (8.33 vs 19.69 and 55.33; *P* < .0001) at 2 months. Similarly, at 3 months, the patients in the experimental group had statistically significantly lower scores compared to control group 1 in pain (1.89 vs 5.69; *P* < .0001), stiffness (0.33 vs 1.44; *P* = .0002), physical function (5.06 vs 16.06; *P* < .0001), and total function (7.28 vs 23.19; *P* < .0001) (see Table 3).

**Table 3:** Comparisons of WOMAC function score information of patients with knee osteoarthritis among study group (per-protocol approach).

WOMAC function score (at 2 mo)	Study groups			*P* (2-sided)	Pairwise mean diff (95% CI)
	Second control (n = 15)–G3	Control (n = 16)–G2	Experimental (n = 18)–G1		
Pain score	12.7 (2.45)	4.81 (1.83)	2.22 (1.48)	<.0001	G3 vs G1 10.64 (9.29–12.00)-*P* < .0001 G3 vs G2 8.05 (6.66–9.45)-*P* < .0001 G2 vs G1 2.59 (1.26–3.92)-*P* = .0003
Stiffness score	4.33 (0.90)	1.56 (0.96)	0.39 (0.70)	<.0001	G3 vs G1 3.94 (3.34–4.55) *P* < .0001 G3 vs G2 2.77 (2.15–3.39)-*P* < .0001 G2 vs G1 1.17 (0.58–1.76)-*P* = .0002
Physical function score	38.13 (6.56)	13.31 (4.51)	5.72 (3.74)	<.0001	G3 vs G1 32.41 (28.90–35.92)-<.0001 G3 vs G2 24.82 (21.21–28.43)- <.0001 G2 vs G1 7.59 (4.14–11.04)- <.0001
Total WOMAC score	55.33 (8.85)	19.69 (6.42)	8.33 (5.05)	<.0001	G3 vs G1 47.00 (42.1951.81) *P* < .0001 G3 vs G2 35.65 (30.7040.59) *P* < .0001
At 3 mo					
Pain score		5.69 (2.06)	1.89 (1.60)	<.0001	NA
Stiffness score		1.44 (0.89)	0.33 (0.59)	.0002	NA
Physical function score		16.06 (4.23)	5.06 (3.65)	<.0001	NA
Total WOMAC score		23.19 (6.24)	7.28 (5.17)	<.0001	NA

ANOVA 1-way was performed for statistical analyses. The post hoc comparisons were performed using a Tukey test.

WOMAC = Western Ontario and McMaster Universities Osteoarthritis Index.

### 3.2. Comparisons of TUG scores per-protocol approach

The patients in the experimental group had statistically significantly lower balance scores compared to the first and second control groups at 2 months, 8.56 versus 9.30 and 14.22; *P* < .0001, and they had statistically significantly lower balance scores compared to the control group at 3 months, 8.71 versus 10.25; *P* = .0164 (see Table 4).

**Table 4:** Comparisons of TUG score information of patients with knee osteoarthritis among study group (per-protocol approach).

TUG score	Study groups			*P* value (2-sided)	Pairwise
	Second control (n = 15)–G3	Control (n = 16)–G2	Experimental (n = 18)–G1		
TUG (baseline)	15.50 (2.61)	15.18 (3.62)	14.42 (1.83)	.5094	Not applicable
TUG (2 mo)	14.22 (2.86)	9.30 (1.50)	8.56 (1.49)	<.0001	G3 vs G1 (*P*=<.0001) G3 vs G2 (*P*=<.0001)
TUG (3 mo)		10.25 (1.94)	8.71 (1.61)	.0164	G1 vs G2 (*P* = .0164)

ANOVA 1-way was performed for statistical analyses. The pairwise comparisons were examined in the Tukey test.

TUG = timed up and go test.

### 3.3. Pre- and post-comparisons of WOMAC scores of the second control group

The pain score was a statistically significant decrease from baseline to 2 and 3 months, from 14.13 to 5.69 and 14.13 to 5.69, respectively. The stiffness score was also a statistically significant decrease from baseline to 2 and 3 months, from 4.81 to 1.56 and 4.81 to 1.44, respectively. Likewise, physical function scores saw a statistically significant decrease from baseline to 2 and 3 months, from 42.06 to 13.31 and 42.06 to 23.19. And the total WOMAC score saw a statistically significant decrease from baseline to 2 and 3 months, from 61.00 to 19.69 and 61.00 to 23.19, respectively. However, regarding the physical function score, there was a significant difference between 2 and 3 months, and the score increased from 13.31 to 23.19 (*P* = <.0001) (Table 5).

**Table 5:** Comparisons of WOMAC score of patients with knee osteoarthritis between study steps (per-protocol approach) in the control groups.

WOMAC scores	Mean	Mean Diff (95% CI)	*P* value (2-sided)
Pain score Pain score (2 mo) Pain score (baseline)	5.69 14.13	−8.44 (−7.15 to −9.73)	<.0001
Pain score (3 mo) Pain score (baseline)	5.69 14.13	−8.44 (−7.15 to −9.73)	<.0001
Pain score (3 mo) Pain score (2 mo)	5.69 5.69	0 (NA)	NA
Stiffness score			
Stiffness score (2 mo) Stiffness score (baseline)	1.56 4.81	−3.25 (−2.75 to −3.75)	<.0001
Stiffness score (3 mo) Stiffness score (baseline)	1.44 4.81	−3.38 (−2.60 to −4.15)	<.0001
Stiffness score (3 mo) Stiffness score (2 mo)	1.44 1.56	−0.125 (0.39 to −0.64)	.6091
Physical function score			
Physical function score (2 mo) Physical function score (baseline)	13.31 42.06	−28.75 (−25.51 to −31.99)	<.0001
Total WOMAC score (3 mo) Physical function score (baseline)	23.19 42.06	−18.88 (−14.86 to −22.89)	<.0001
Total WOMAC score (3 mo) Physical function score (2 mo)	23.19 13.31	9.88 (13.13–6.62)	<.0001
Total WOMAC score			
Total WOMAC score (2 mo) Total WOMAC score (baseline)	19.69 61.00	−41.31 (−36.57 to −46.06)	<.0001
Total WOMAC score (3 mo) Total WOMAC score (baseline)	23.19 61.00	−37.81 (−33.13 to −42.50)	<.0001
Total WOMAC score (3 mo) Total WOMAC score (2 mo)	23.19 19.69	3.5 (7.06 to −0.06)	0.0536

Bonferroni correction was performed for statistical analyses.

WOMAC = Western Ontario and McMaster Universities Osteoarthritis Index.

### 3.4. Pre- and post-comparisons of WOMAC scores of the experimental group

The pain score showed a statistically significant decrease from baseline to 2 and 3 months, from 13.16 to 1.88 and 13.16 to 1.88, respectively. The stiffness score decreased statistically from baseline to 2 and 3 months, from 4.88 to 0.38 and 4.88 to 0.33. In addition, there was a statistically significant decrease in physical function scores from baseline to 2 and 3 months, from 38.38 to 5.72 and 38.38 to 5.05. The total WOMAC score had a statistically significant decrease from baseline to 2 and 3 months, from 56.44 to 8.33 and 56.44 to 7.27 (See Table 6). No complications were found in any patients of the 3 groups after 2 and 3 months.

**Table 6:** Comparisons of WOMAC score information of patients with knee osteoarthritis between study steps (per-protocol approach) in the experimental group.

WOMAC score	Mean	Mean Diff (95% CI	*P* value (2-sided)
Pain score			
Pain score (2 mo) Pain score (baseline)	1.89 13.17	−11.278 (−10.22 to −12.34)	<.0001
Pain score (3 mo) Pain score (baseline)	1.89 13.17	−11.278 (−10.22 to −12.34)	<.0001
Pain score (3 mo) Pain score (2 mo)	1.89 1.89	0 (NA)	NA
Stiffness score			
Stiffness score (2 mo) Stiffness score (baseline)	0.39 4.89	−4.5 (−3.90 to −5.10)	<.0001
Stiffness score (3 mo) Stiffness score (baseline)	0.33 4.89	−4.56 (−3.96 to −5.15)	<.0001
Stiffness score (3 mo) Stiffness score (2 mo)	0.33 0.39	−0.06 (0.26 to −0.37)	.7168
Physical function score			
Physical function score (2 mo) Physical function score (baseline)	5.72 38.39	−32.67 (−29.28 to −36.06)	<.0001
Physical function score (3 mo) Physical function score (baseline)	5.06 38.39	−33.33 (−30.36 to −36.31)	<.0001
Physical function score (3 mo) Physical function score (2 mo)	5.06 5.72	−0.67 (0.46 to −1.80)	.2307
Total WOMAC score			
Total WOMAC score (2 mo) Total WOMAC score (baseline)	8.33 56.44	−48.11 (−43.35 to −52.87)	<.0001
Total WOMAC score (3 mo) Total WOMAC score (baseline)	7.28 56.44	−49.17 (−45.01 to −53.32)	<.0001
Total WOMAC score (3 mo) Total WOMAC score (2 mo)	7.28 8.33	−1.06 (0.46 to −2.57)	.1588

Bonferroni correction was performed for statistical analyses.

WOMAC = Western Ontario and McMaster Universities Osteoarthritis Index.

## 4. Discussion

To the best of our knowledge, this is the first study that examined the effectiveness of HBE with cryotherapy on functional improvement in patients with KOA. The study showed at 2 and 3 months that improvements in pain, stiffness, difficulty performing a physical function, and balance were associated with HBE along with cryotherapy compared to those patients who received HBE intervention only or no intervention.

The effectiveness of HBE, along with cryotherapy, on the functions of patients with KOA, has yet to be examined previously. However, the main effect of this intervention may return to cryotherapy. We applied cryotherapy with HBE intervention to patients because leaving them without physiotherapy interventions is unethical.

The effectiveness of cryotherapy on 60 KOA patients was evaluated in a randomized controlled trial (RCT). Incidentally, 30 patients received cryotherapy, delivered as packs of crushed ice in 2 plastic bags (24 × 3 34 × 3 × 0.08 cm), each containing 1 kg of crushed ice. The bags of crushed ice were placed on the knee, covering the medial, posterior, and lateral surfaces, and applied with mild compression for 20 minutes once a day for 4 consecutive days. Instead of crushed ice, the control group used sand-filled sham packs. They did not report a significant difference in pain between the cryotherapy and sham study groups, but function and quality of life were improved.^[24]^ The Ottawa Panel, the European League Against Rheumatism, and the International Osteoarthritis Research Society failed to agree among the expert panel. They left cryotherapy out of their final recommendations.^[17,25]^ They suggested that cryotherapy may not be beneficial as a treatment for KOA. However, the American College of Rheumatology and the National Institute for Health and Care Excellence have conditionally advised cryotherapy as an additional treatment option for persons with KOA.^[16]^

Another clinical trial compared to exercise, cryotherapy, and short-wave therapy in treating KOA grade I. There were 25 participants, ages 58 to 78. For 20 minutes, Group A exercised and received short waves. Group B performed workouts while using ice for 20 minutes, and Group C only exercised. All patients underwent physical therapy at an outpatient facility for ten sessions twice a week, using isometry (stretching and strengthening exercises) for the hip abductors, sural triceps, adductors, iliotibial, and quadriceps, an ergometric bicycle with a proprioception cushion, and, depending on the group, application of short waves or ice. The study concluded that the most appropriate pain relief protocol was a combination of ice and kinesiotherapy. Their research also revealed that using only kinesiotherapy did not affect pain but did improve functional quality, muscular strength, and range of motion.^[26]^

Another RCT compared the efficacy of manual therapy combined with cryotherapy to kinesiotherapy combined with cryotherapy for treating KOA. Cryotherapy and manual therapy were administered to the intervention group (n = 64). The knee joint was subjected to cryotherapy using a cold nitrogen vapor of just 130°C. The patient body surface was 10 cm from the equipment nozzle, and no longer than 3 minutes were allowed. The manual treatment techniques included tibiofemoral distraction, anterior tibial glide, posterior tibial glide, medial and lateral tibia rotation mobilization, and patellofemoral glides.^[27]^

Each session was, at most, 45 minutes. The control group received kinesiotherapy, warming up for 5 minutes, stretching for 5 minutes, strengthening for 15 minutes, and stationary cycling for 5 minutes, combined with cryotherapy (KIN-C group). The study reported that manual therapy combined with cryotherapy reduced pain and improved the quality of life, range of motion, and functional exercise capacity than kinesiotherapy with cryotherapy.^[28]^ Our findings agreed with this study regarding the benefits of cryotherapy, but we do not prefer to use cryotherapy in the first steps to prevent additional injury. Since ice directly affects the tendinous body and muscle spindle, overexertion following muscle cooling might result in a new muscle injury because the motor control threshold is alert.^[29]^

A systematic review of 3 RCTs^[30]^ was performed, considering varied designs, outcomes measured, thermotherapy or cryotherapy treatments, and the overall methodological quality. The study measured ice massage effectiveness in 1 trial compared to the control group. Twenty-minute bouts of ice massage, 5 days a week for 2 weeks, led to a clinically significant benefit in increasing quadriceps strength (29% relative difference) compared with the control group.^[31]^

Furthermore, the systematic review included a study that investigated the impact of ice packs on pain when compared to the control group. The treatment with ice packs or control (undetermined period of therapy) was taken 3 sessions a week for 3 weeks. A difference that was almost statistically significant was found, according to the study findings (weighted mean difference = −2.70, 95% CI: −5.52 to 0.12; *P* = .06) between the control group and ice pack group after 3 weeks of treatment. However, the 2 groups detected no significant differences in pain at the 3-month follow-up.^[32]^

The third trial measured the efficacy of hot or cold packs compared to the control group. The application of hot or cold (10 sessions of 20 minutes) on the posterior and anterior of the affected knee was measured. The results showed knee circumference edema reduction (*P* = .04) after ten cold treatment sessions. For the application of hot packs, no significant effects were recorded when compared to the control group.^[33]^ Finally, the systematic review concluded that the ice massage significantly benefited knee strength, range of motion, and function compared to the control group. It can be concluded that cold packs reduced knee swelling, while hot packs had no significant effects on edema compared with cold or placebo applications. The pain of patients with OA was not affected by ice packs compared to the control group.

In our study, we combined HBE with cryotherapy to evaluate the effects of cryotherapy. The HBE was taken from a previous quasi-experimental study that assessed the impact of HBE on 141 patients (60 years of age or older) with KOA.^[28]^ The experimental group received HBE and health education, and the control groups received health education for 12 months. The outcomes of their study, including pain intensity and joint stiffness related to KOA, were measured by the WOMAC questionnaire.^[22]^ The results showed that HBE and health education reduced pain intensity from 7.34 (3.36) to 4.28 (3.30), joint stiffness from 2 (0.3) to 1 (0.3), and increased the muscle strength of the lower limbs from 14.22 (3.10) to 12.13 (2.93), balance from 13.30 (3.14) to 11.73 (1.97), mobility from 408.45 (60.54) to 442.39 (49.70), and improved the quality of life of the participants. Similar to that study, our results showed a highly significant effect of HBE combined with cryotherapy in treated groups compared with the control groups in improving pain intensity, joint stiffness, daily function and balance.

After 2 months of intervention, the patients who obtained cryotherapy and HBE were significantly better than the control groups. One month after the intervention (without treatment), we measured the effect of cryotherapy and HBE. The effect was visible and significantly better than the control groups regarding pain intensity, joint stiffness, daily function, and balance. These improvements may be attributed to the effect of cryotherapy. However, there may be some hidden confounders that interact with this effect.

Cryotherapy may reduce pain and inflammation, as well as local metabolism, which lowers cellular energy needs and, as a result, reduces secondary hypoxic tissue injury.^[34]^ Fluid filtration into the interstitial tissue may be reduced with cold therapy due to vasoconstriction and the prevention of dramatic increases in microvascular permeability.^[35]^ Evidence suggests that cryotherapy helps pain reduction, swelling, and inflammation in KOA. Adding cryotherapy to manual therapy or kinesiotherapy may enhance the health benefits in patients with KOA.^[36,37]^ Cryotherapy seems to reduce synovial inflammation due to inflammatory cytokine concentration reduction and lower leukocyte migration to the knee joint cavity in the rat model of post-traumatic KOA.^[38]^

### 4.1. Strengths and limitations of the study

We tried to include a representative population sample in this study as much as possible. In this regard, we obtained the required general and medical information of patients with KOA before recruitment and interventions. In addition, we successfully performed a randomization process, combined our efforts to reduce the number of lost-to-follow-up patients, and adhered to the inclusion and exclusion criteria. In this way, we excluded patients with other possible effects on pain and function. However, the study was not exempt from limitations because we could not include patients with higher BMIs. The current effects can be applied to patients with higher BMIs as well. However, the findings reported in this study may not be applied or less applied to patients in other clinical settings due to different exercises or adherence.

### 4.2. Future attempts

The author recommends using crushed ice instead of commercial gel to enhance the effects of cryotherapy for patients with KOA. The researcher also suggests that the effects of cryotherapy should be measured 3 and 6 months after the intervention.

## 5. Conclusions

This study showed that combining HBE and cryotherapy is an effective technique for improving the functions among KOA patients.

## Acknowledgments

The authors would like to thank the participants who participated in this study.

## Author contributions

**Conceptualization:** Hawar A. Mohammed Sadiq, Mohammad Tahir Rasool.

**Data curation:** Hawar A. Mohammed Sadiq.

**Formal analysis:** Hawar A. Mohammed Sadiq, Mohammad Tahir Rasool.

**Funding acquisition:** Hawar A. Mohammed Sadiq.

**Investigation:** Hawar A. Mohammed Sadiq.

**Methodology:** Hawar A. Mohammed Sadiq.

**Project administration:** Hawar A. Mohammed Sadiq, Mohammad Tahir Rasool.

**Supervision:** Mohammad Tahir Rasool.

**Validation:** Mohammad Tahir Rasool.

**Writing – original draft:** Hawar A. Mohammed Sadiq.

**Writing – review & editing:** Mohammad Tahir Rasool.

## Supplementary Material


