# Supplementary material for: Effectiveness of home-based conventional exercise and cryotherapy on daily living activities in patients with knee osteoarthritis: A randomized controlled clinical trial
Source: Medicine (Baltimore). 2023 May 5;102(18):e33678. doi: 10.1097/MD.0000000000033678 (PMC10158910; doi:10.1097/MD.0000000000033678)
Supplement: Supplementary file 1 [file medi-102-e33678-s001.pdf]

Supplementary: Details of home-based exercises & cryotherapy

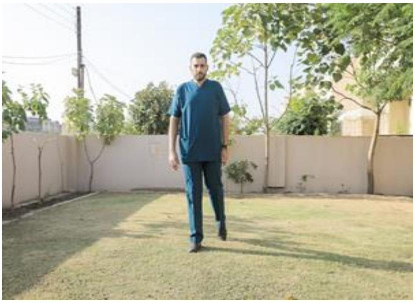

Warm-up walking for 5 minutes

A： Passive knee flexion

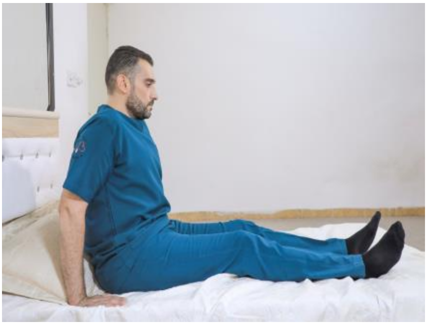<br>①Sit on the bed	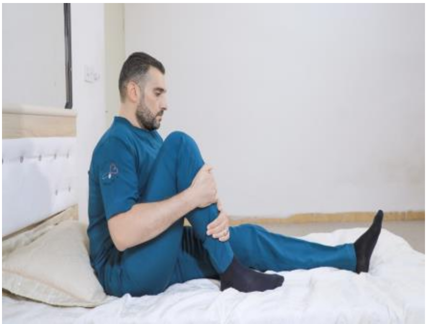<br>②Hold your hands on one side of the ankle, slowly and forcefully hold the leg to the chest to maximize knee flexion, keep 60 seconds
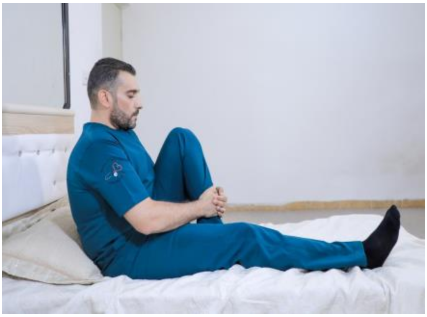<br>③Relax this leg and repeat the above	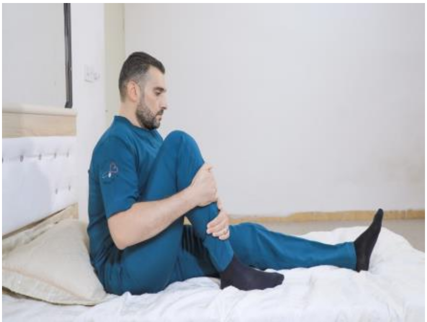<br>④Repeat the exercise 3 times with

action on the other side	both legs

B：Passive knee extension

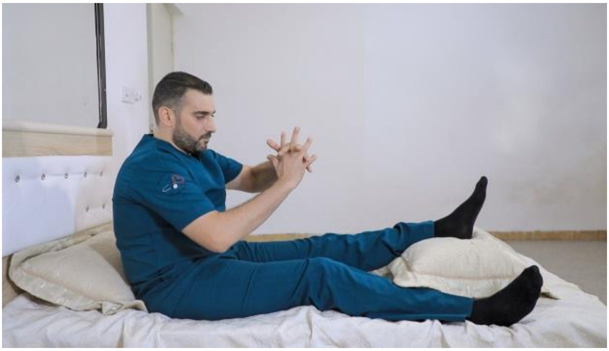<br>①Sit on the bed; Put one side of the foot pad 8~10 cm high	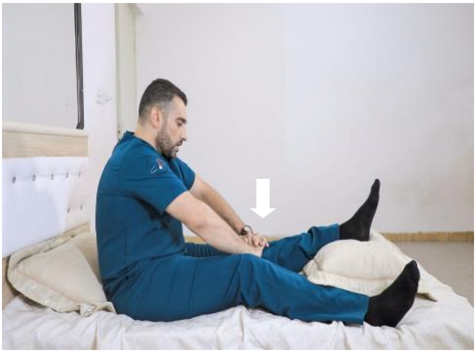<br>②Apply light weight to the raised knee joint or apply proper pressure by hand for 60 seconds
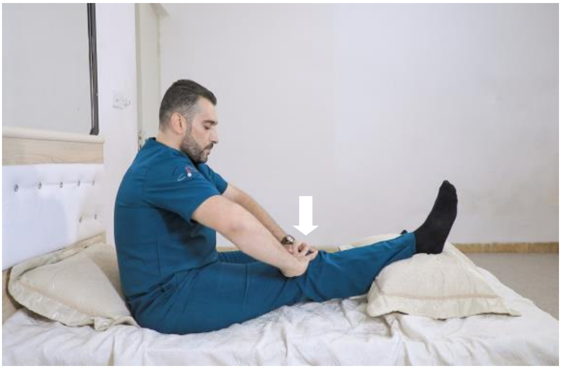<br>③Relax this leg and repeat the above action on the other side	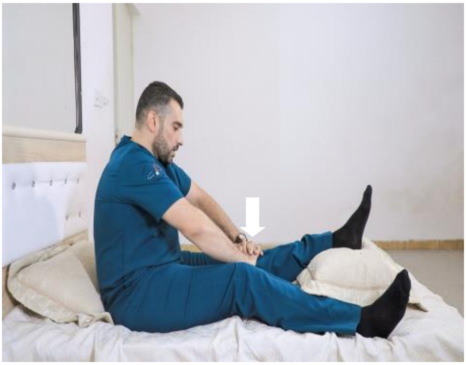<br>④Repeat the exercise 2 times with both legs

C： Isometric contractions of the quadriceps

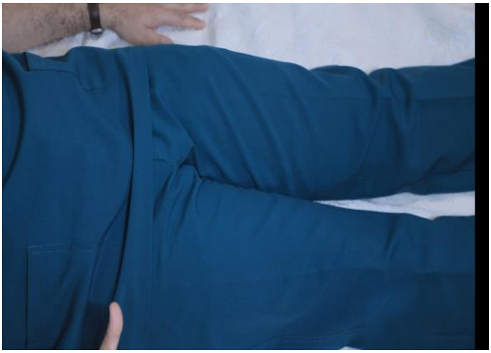<br>①Sitting or lying down, legs relaxing	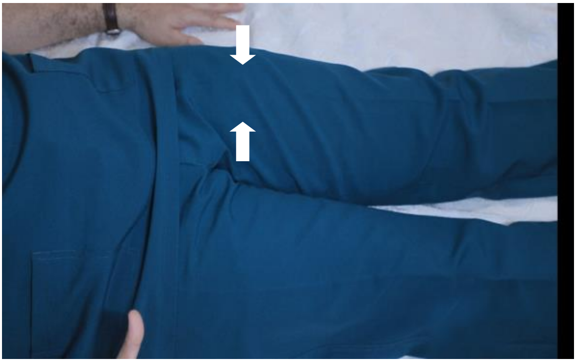<br>② Tight the thigh muscles on one side with maximum strength, keep it for 5 seconds, and relax for 2 seconds. Repeat 10 times for 1 group and practice 10 groups in succession
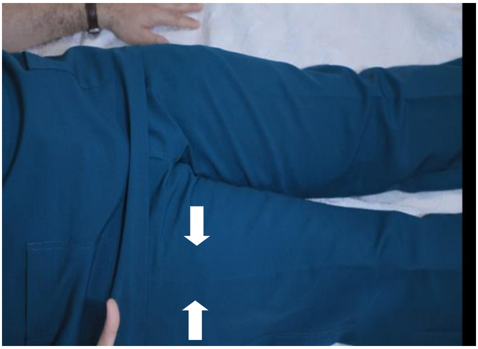<br>③ Relax this leg and repeat the above action on the other side	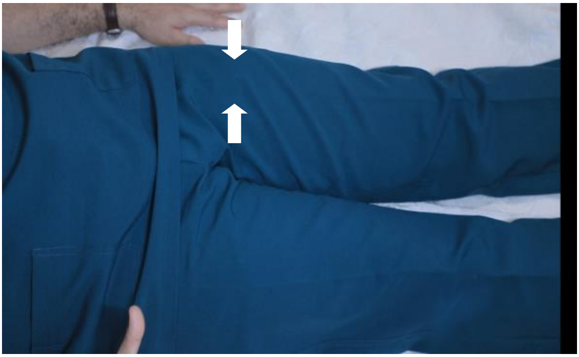<br>④Repeat the exercise 4 times with both legs

D：Supine straight-leg lifts

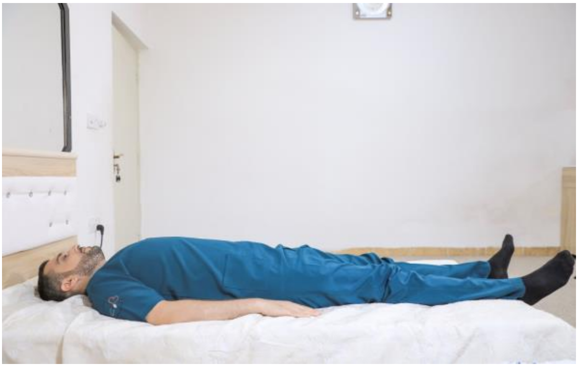<br>①lie on the back, stretch knees	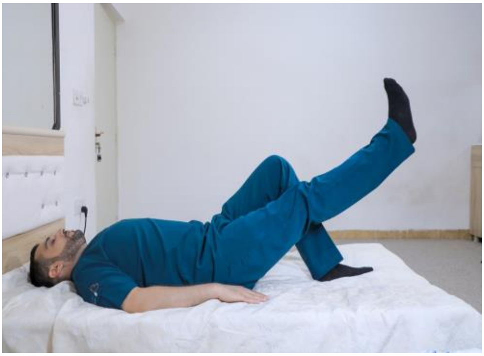<br>②One leg is flexed to support the bed surface, the other leg is raised to the heel, about 20 cm away from the bed, held for 5 seconds, put down for 5 seconds, repeat 10 times
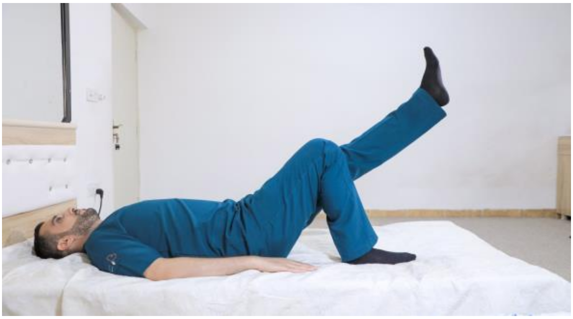<br>③Relax this leg and repeat the above action on the other side	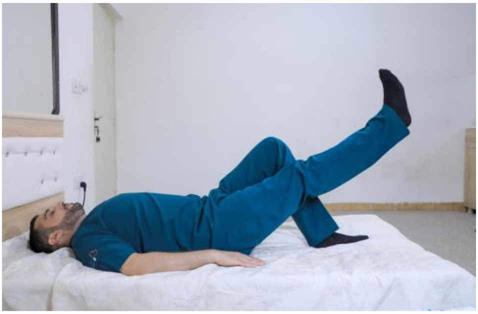<br>④Repeat the exercise 3 times with both legs

E：Leg lifts in the prone position

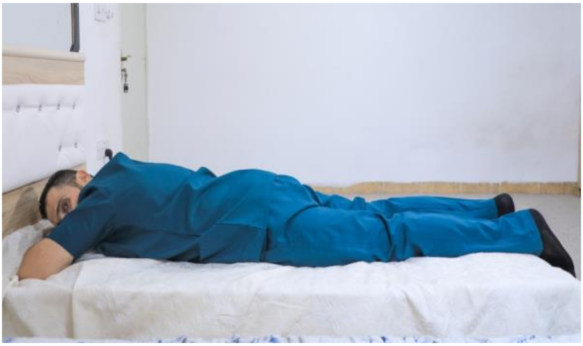 ①Lie face down, stretch knees	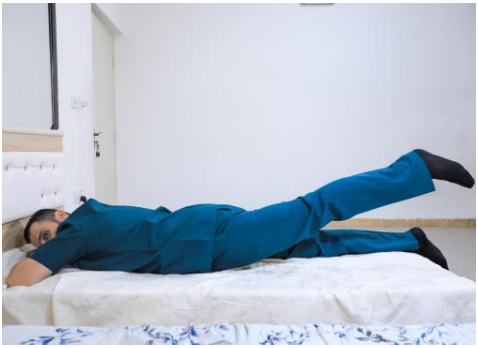 ②Lift one leg back to the toe, about 20 cm away from the bed, held for 5 seconds, put down for 5 seconds, repeat 10 times
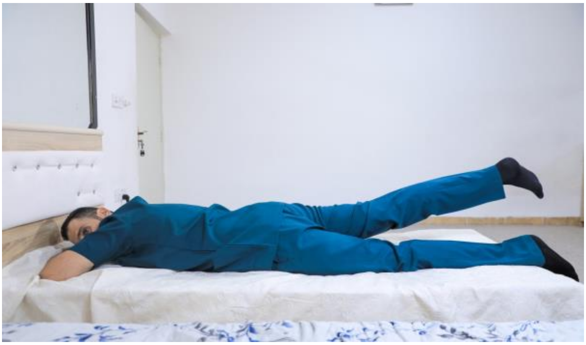 ③Relax this leg and repeat the above action on the other side	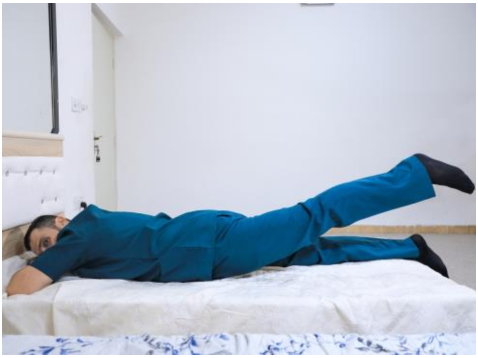 ④Repeat the exercise 3 times with both legs

F： Shifting the center of gravity (left and right)

<table>
<tr><td>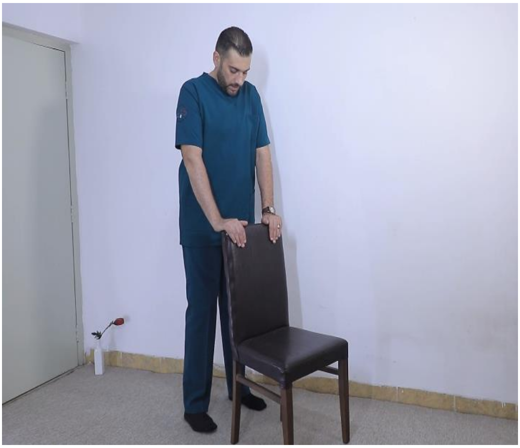
①Stand up and support a chair with a height of 70~80 cm and open the feet</td><td>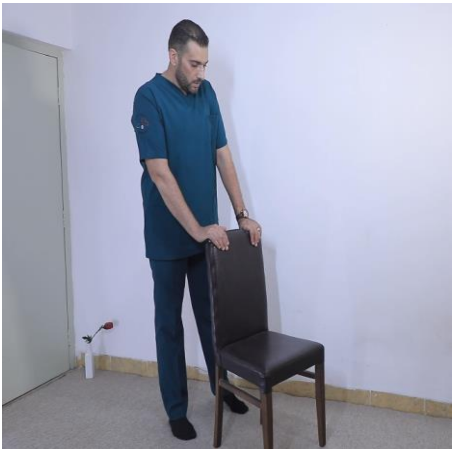
②Keep your knees upright, slowly move the center of gravity to the left, and gradually lower your right heel</td></tr>
<tr><td>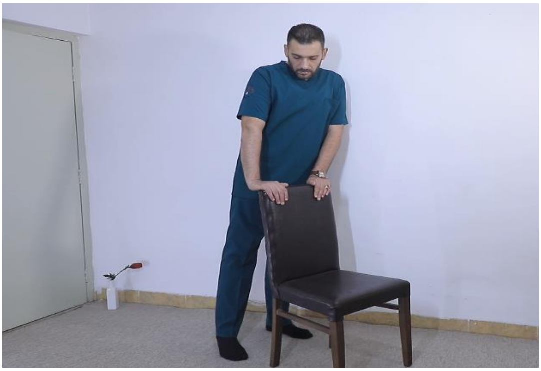
③Keep your knees upright, slowly move the</td><td>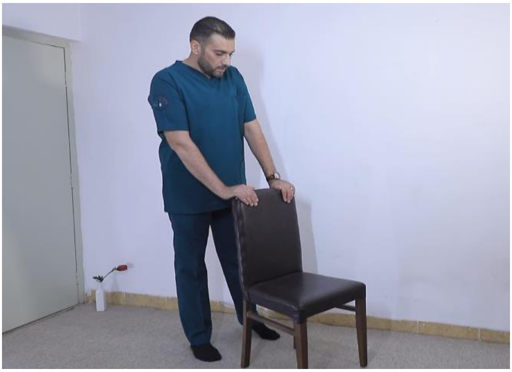
④Repeat the above action for 3 minutes</td></tr>
</table>

center of gravity to the right, and gradually lower your left heel	

G： Shifting the center of gravity (forwards and backwards)

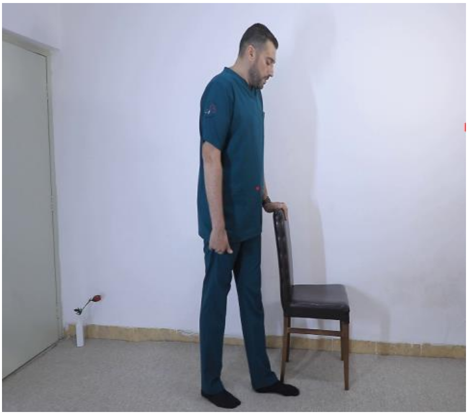	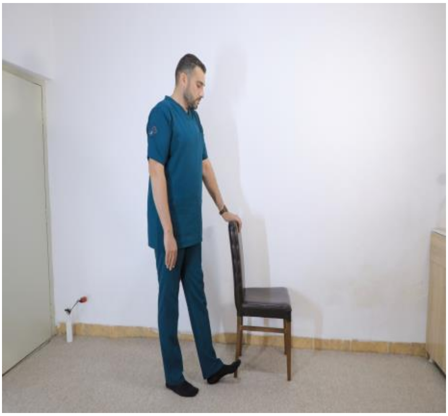
①Stand up and support a chair with a height of 70~80 cm and take one step forward on one side	②Keep the knees upright, slowly move the center of gravity forward, and the heel of the hind foot gradually leaves the ground
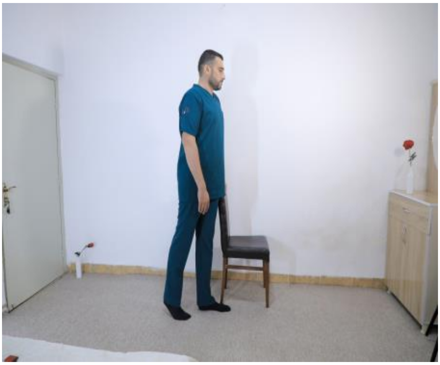	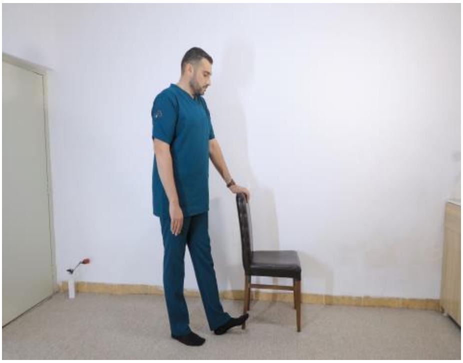

③Keep the knees upright, slowly move the center of gravity backwards, and the forefoot gradually leaves the ground	④Repeat the above action for 3 minutes

H：Resistance knee extension

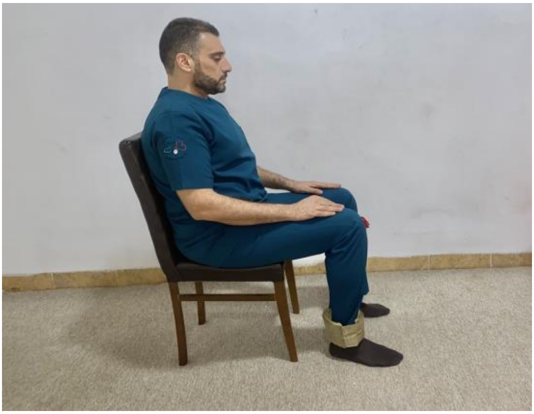<br>①Sit on the chair or at the bed, tie a 1kg sandbag to the ankle, keep the upper body straight	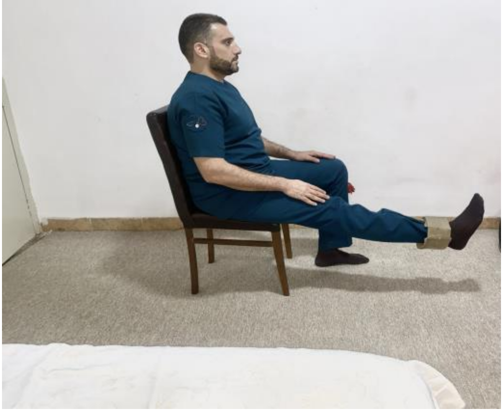<br>②Do not move the thighs, lift your calves until the knees are fully extended, hold for 5 seconds, rest your legs for 5 seconds, repeat 10 times
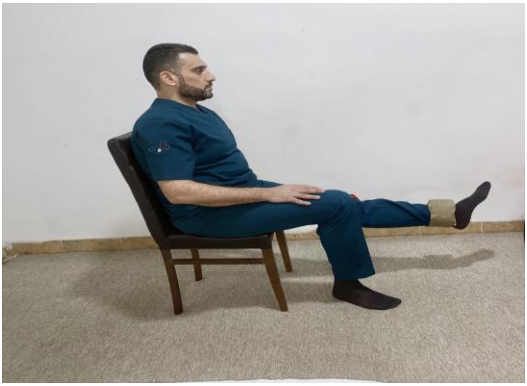	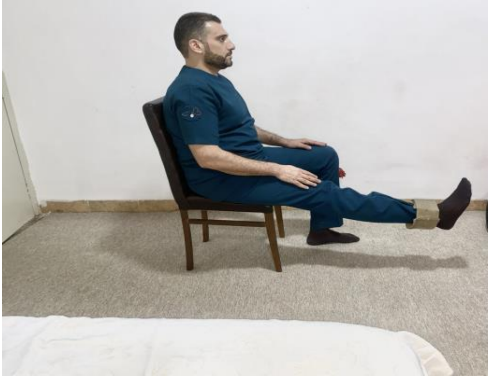

③Relax this leg and repeat the above action on the other side	④Repeat the exercise 3 times with both legs

I： Resistance knee flexion

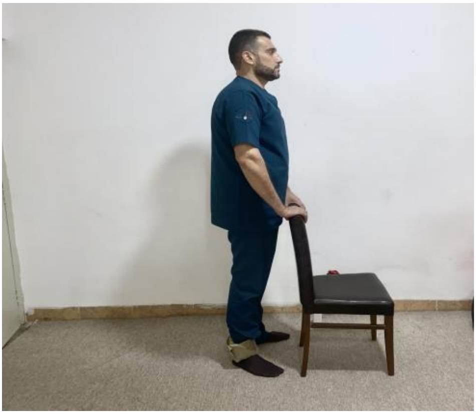	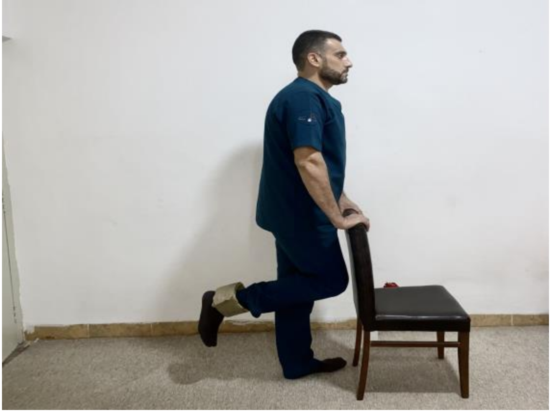
① Standing up, tie a 1kg weight sandbag to the ankle joint, and support the upper edge of the chair	② Stand on one leg and pull the calf back to the other leg, flexing the knee as much as possible while keeping the thigh perpendicular to the ground. Hold for 5 seconds, put your legs down for 5 seconds, repeat 10 times

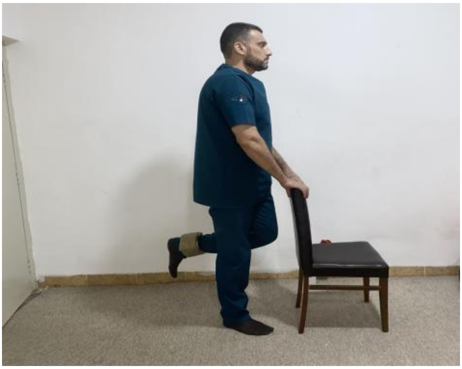 ③ Relax this leg and repeat the above action on the other side	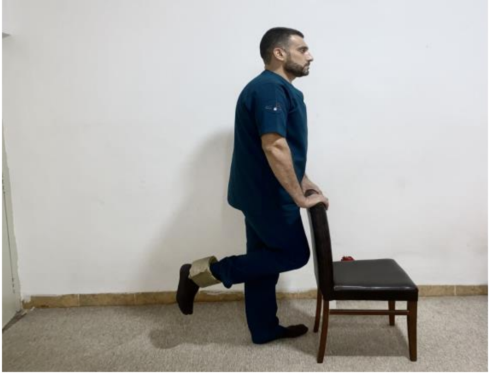 ④Repeat the exercise 3 times with both legs

J- Cryotherapy by using Reusable gel Ice packs

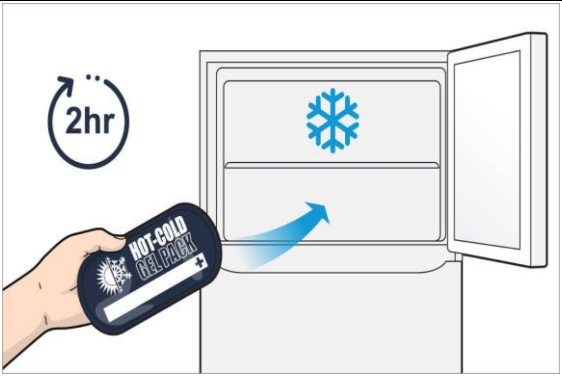 ① Place gel pack in freeze for 2 hours minimum for best result	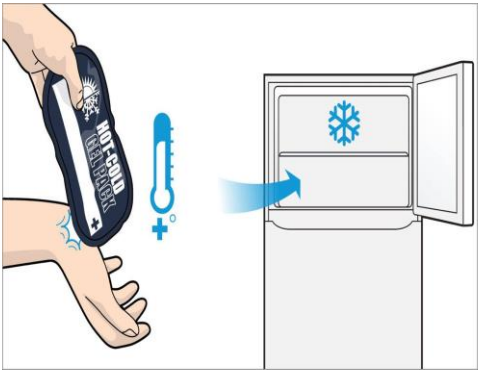 ② Touch-test the temperature for any allergies to cryotherapy

Supplementary: Details of home-based exercises & cryotherapy

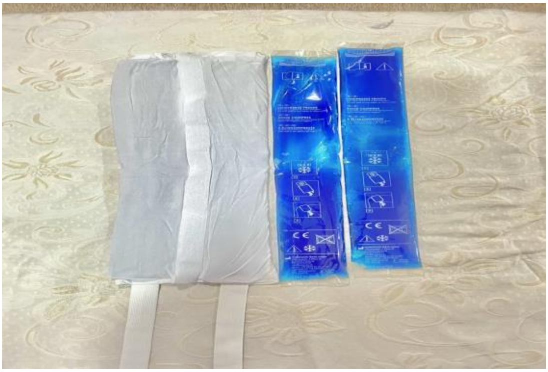<br>③ Put the reusable gel in its cover before applying to the knee	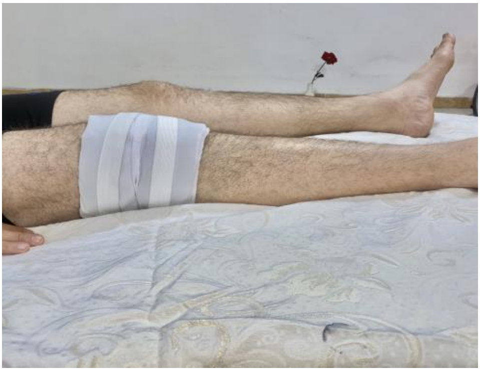<br>④Two packs of reusable gel ice packs placed on the knee for 20 min, covering the anterior, posterior, medial, and lateral surfaces of the knee.
